# Integrative Metabolomic Evidence of Bioactive Food-Derived Compounds Targeting Alzheimer’s Disease: A Cross-Study Analysis in *Caenorhabditis elegans*

**DOI:** 10.3390/ijms27156591

**Published:** 2026-07-24

**Authors:** Tamara Y. Forbes-Hernández, Carmen Quiles-Ramírez, Francesca Giampieri, Justyna Godos, Giuseppe Grosso, Carmen L. Rodríguez-Velasco, Jianbo Xiao, Maowen Ding, Maurizio Battino, Lorenzo Rivas-García, Cristina Sánchez-González

**Affiliations:** 1 Departimento di Scienze Biomediche, University of Cagliari, Cittadella Universitaria—S.S. 554 Bivio per Sestu, 09042 Monserrato, Italy; 2 Andalusian Research Institute in Data Science and Computational Intelligence (DaSCI), University of Granada, 18071 Granada, Spain; 3 Dipartimento di Scienze Cliniche Specialistiche, Facoltà di Medicina, Università Politecnica delle Marche, 60131 Ancona, Italy; 4 Research Group on Food, Nutritional Biochemistry and Health, Universidad Europea del Atlántico, 39011 Santander, Spain; 5 Joint Laboratory on Food Science, Nutrition, and Intelligent Processing of Foods, Polytechnic University of Marche, Italy, Universidad Europea del Atlántico Spain and Jiangsu University, China at the Polytechnic University of Marche, 60131 Ancona, Italy; 6 Department of Biomedical and Biotechnological Sciences, University of Catania, 95123 Catania, Italy; 7 Department of Analytical and Food Chemistry, Faculty of Science, University of Vigo, 32004 Ourense, Spain; 8 Department of Physiology, Institute of Nutrition and Food Technology “José Mataix Verdú”, Biomedical Research Centre, University of Granada, Avda. del Conocimiento s.n, 18100 Armilla, Spain; 9 International Joint Research Laboratory of Intelligent Agriculture and Agri-Products Processing, Jiangsu University, Zhenjiang 212013, China; 10 Instituto de Investigación Biosanitaria ibs.GRANADA, 18012 Granada, Spain

**Keywords:** Alzheimer’s disease, antioxidants, phytochemistry, tau protein, paralysis, acetylcholinesterase

## Abstract

Alzheimer’s disease (AD) is a major neurodegenerative condition with limited treatment options. Applications of food-derived products rich in bioactive compounds have emerged as promising strategies, yet comparative evidence across different matrices remains scarce. Here, we present an integrative metabolomic analysis of eleven food extracts previously tested in *Caenorhabditis elegans* models of AD. By combining chemical fingerprints and functional bioassay data—including oxidative stress resistance, β-amyloid-induced paralysis, and tau-associated locomotion—we employed multivariate statistics to uncover common patterns and correlations. Principal Component Analysis, PLS-DA, and hierarchical clustering revealed distinct groupings of extracts based on metabolite profiles and neuroprotective effects. Polyphenols, flavonoids, and iridoids were consistently associated with beneficial outcomes, while antioxidant capacity and acetylcholinesterase inhibition emerged as key functional traits. Correlation heatmaps highlighted extract-specific strengths, suggesting potential for rational combinations targeting complementary bioactivities. This work underscores the value of integrating metabolomics and functional biology to inform the design of food-based interventions for neurodegenerative diseases, paving the way for evidence-based innovation in brain health.

## 1. Introduction

Neurodegenerative disorders, particularly Alzheimer’s disease (AD), represent one of the major global health challenges of the twenty-first century [1,2]. The prevalence of dementia is rising rapidly in aging populations, and current estimates suggest that over 55 million people are affected worldwide—a number projected to double by 2050 [3,4]. Beyond the clinical burden, dementia imposes substantial economic and social costs on families and healthcare systems [4,5]. Despite intense pharmaceutical efforts, existing treatments provide only symptomatic relief and fail to halt disease progression [6]. Therefore, identifying complementary or preventive strategies that can modulate disease mechanisms at earlier stages has become a priority in neurodegenerative research [7].

Accumulating evidence supports the role of diet and bioactive food compounds in preserving cognitive function and mitigating neurodegenerative processes [8,9]. Several epidemiological and clinical studies have linked adherence to dietary patterns such as the Mediterranean diet with reduced risk of cognitive decline [10,11]. Specific bioactive molecules—polyphenols, flavonoids, phenolic alcohols, and omega-3 fatty acids—have been reported to exert antioxidant, anti-inflammatory, and proteostatic effects relevant to Alzheimer’s pathology [12,13]. However, clinical outcomes remain heterogeneous due to variations in extract composition, dosing, and study design [14]. This highlights the need for integrative approaches that connect molecular mechanisms with consistent biological outcomes across models and compound families.

In the preclinical domain, *Caenorhabditis elegans* has emerged as a powerful model organism for investigating neurodegenerative processes and testing bioactive compounds [15,16]. Its conserved molecular pathways related to oxidative stress, protein aggregation, and longevity make it particularly suitable for mechanistic studies [17]. Over the past five years, our group has systematically employed *C. elegans* to evaluate the neuroprotective potential of food-derived products and by-products from the agri-food industry, including olive [18,19,20], garlic [21], broccoli [22], strawberry [23] and edible flower derivatives [24,25,26], as well as honey varieties [27,28]. These studies shared a common methodological framework, combining phenotypic assays with LC–QTOF-based metabolomic characterization of the tested extracts.

Given the methodological homogeneity and the diversity of tested compounds, the present work aims to integrate data from nine previously published datasets from our laboratory through a meta-analytical re-evaluation using metabolomic tools, i.e., MetaboAnalys [29]. By applying multivariate and clustering analyses in this way, we sought to identify convergent metabolomic profiles and compound families consistently associated with neuroprotective effects in *C. elegans*. This integrative approach could provide a unified view of how food-derived compounds modulate key biochemical pathways implicated in neurodegeneration and support the rational design of multi-component formulations for future translational studies [30]. While the analysis is exploratory in nature, it might illustrate how data harmonization and systems-level metabolomics can uncover reproducible chemical–functional relationships across independent studies, providing a methodological bridge between descriptive metabolomics and hypothesis-driven functional food development.

## 2. Results

The integrative dataset comprised nine independent experimental studies conducted by our group over the past five years (in these studies, eleven individual extracts were evaluated, as one study examined three enrichment levels of olive leaf extract), all employing *Caenorhabditis elegans* as an in vivo model to evaluate the neuroprotective potential of food-derived products and by-products. These studies included extracts or fractions obtained from olive by-products, garlic, broccoli, strawberry, honey varieties, and edible flowers. Each study combined phenotypic assays—such as survival under oxidative stress, β-amyloid aggregation, tau-induced motility defects, and lifespan analysis—with LC–QTOF-based chemical characterization of the tested materials. Despite the diversity of matrices, all experiments followed a comparable methodological framework and assessed convergent biological outcomes related to oxidative stress, proteostasis, and neuroprotection.

### 2.1. Exploratory Multivariate Analysis: K-Means Clustering

**Figure 1:**
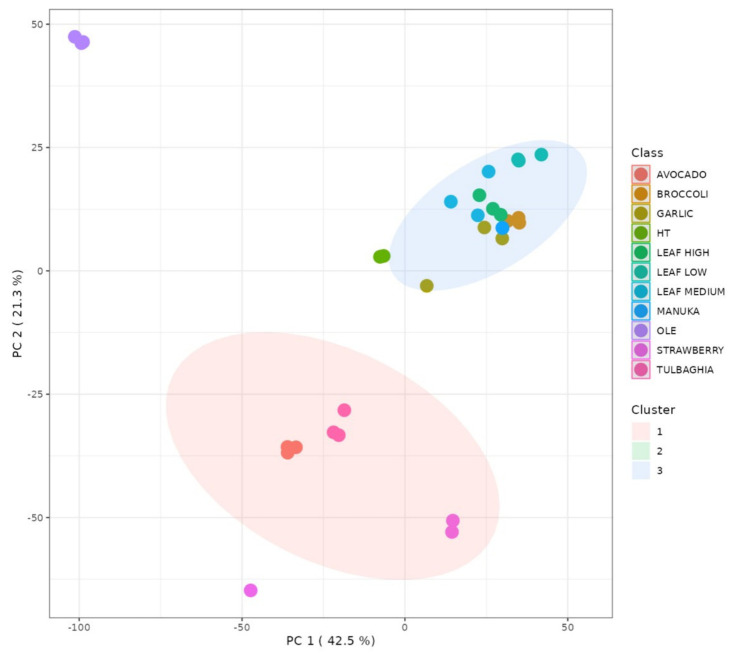
K-means clustering projected onto the first two principal components (PC1 and PC2). Data were auto-scaled and log-transformed prior to analysis in MetaboAnalyst 6.0. Three distinct clusters were identified, grouping the eleven food-derived extracts according to similarities in their combined chemical and functional profiles. Colored ellipses represent the confidence areas for each cluster. Abbreviations and original study references: AVOCADO, avocado honey [28]; BROCOLLI, broccoli by-product extract [22]; GARLIC, garlic extract [21]; HT, hydroxytyrosol-rich extract [19]; LEAF HIGH, olive tree leaf extract high in bioactive compounds [20]; LEAF LOW, olive tree leaf extract low in bioactive compounds [20]; LEAF MEDIUM, olive tree leaf extract with medium bioactive compound content [20]; MANUKA, manuka honey [27]; OLE, olive leaf extract rich in oleuropein [18]; STRAWBERRY, strawberry extract [23]; TULBAGHIA, Tulbaghia extract [25]. Ellipses denote 95% confidence regions for Clusters 1 and 3. Cluster 2, comprising the two OLE replicates, is not delimited by an ellipse due to the insufficient sample size (n = 2) required for ellipse estimation.

To explore the intrinsic organization of the dataset, an unsupervised k-means clustering analysis was conducted based on metabolomic composition. The optimal number of clusters, determined by the silhouette and elbow criteria, was three, capturing 63.8% of total variance across the first two principal components (Figure 1). Cluster 1 comprised honey and fruit-derived matrices—avocado honey, manuka honey, and strawberry—together with *Tulbaghia*, which displayed a partially overlapping metabolomic signature possibly driven by shared low-polarity phenolics and volatile compounds. Cluster 2 included all olive-derived products (olive leaf extracts with low, medium, and high enrichment; hydroxytyrosol; and OLE), reflecting their homogeneous composition in secoiridoids and phenolic alcohols. Cluster 3 grouped broccoli and garlic, characterized by glucosinolate- and sulfur-containing metabolites. The reproducible and chemically coherent clustering pattern indicates that samples from related biological sources display consistent metabolomic fingerprints, supporting the internal coherence of the integrated dataset. These patterns should nonetheless be regarded as exploratory, given that the underlying dataset was built from reconstructed triplicates.

### 2.2. Supervised Classification and Variable Importance

#### 2.2.1. Partial Least Squares Discriminant Analysis (PLS-DA)

**Figure 2:**
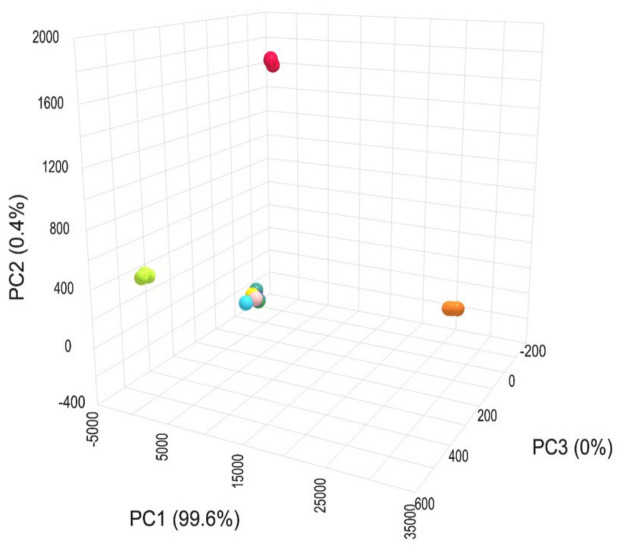
Three-dimensional Partial Least Squares Discriminant Analysis (PLS-DA) score plot showing the distribution of the eleven food-derived extracts based on their integrated chemical and biological profiles. The first three latent components explain most of the model variance, with component 1 (99.6%) driving the main separation among groups. The supervised projection highlights distinct clustering patterns consistent with compositional and functional differentiation among matrices.

To confirm and refine the unsupervised patterns observed via k-means clustering, a supervised PLS-DA model was constructed (Figure 2). The first three components explained the total variance structure, with PC1 accounting for 99.6% of the variance and PC2 for 0.4%. The resulting three-dimensional score plot showed a clear and reproducible separation among the different extract groups, consistent with their botanical and compositional origin. Olive-derived samples (LEAF LOW, LEAF MEDIUM, LEAF HIGH, HT, and OLE) clustered tightly together, reflecting their homogeneous content in secoiridoids and phenolic alcohols. In contrast, honey- and fruit-based matrices (avocado honey, manuka honey, strawberry, and broccoli) occupied an opposite region along PC1, characterized by higher representation of flavonoids, phenolic acids, and carbohydrate-derived metabolites. Sulfur-rich samples (garlic and *Tulbaghia*) formed a distinct cluster separated from the rest, indicating a metabolomic signature dominated by organosulfur compounds. The well-defined group separation is consistent with distinct chemical fingerprints among the different classes of food-derived compounds. However, since the model was built on reconstructed triplicates derived from mean ± SE values, these results should be interpreted as hypothesis-generating rather than confirmatory. No permutation testing or cross-validation was performed; the model was used here in an exploratory capacity to guide the subsequent VIP analysis.

#### 2.2.2. Variable Importance in Projection (VIP) Analysis

**Figure 3:**
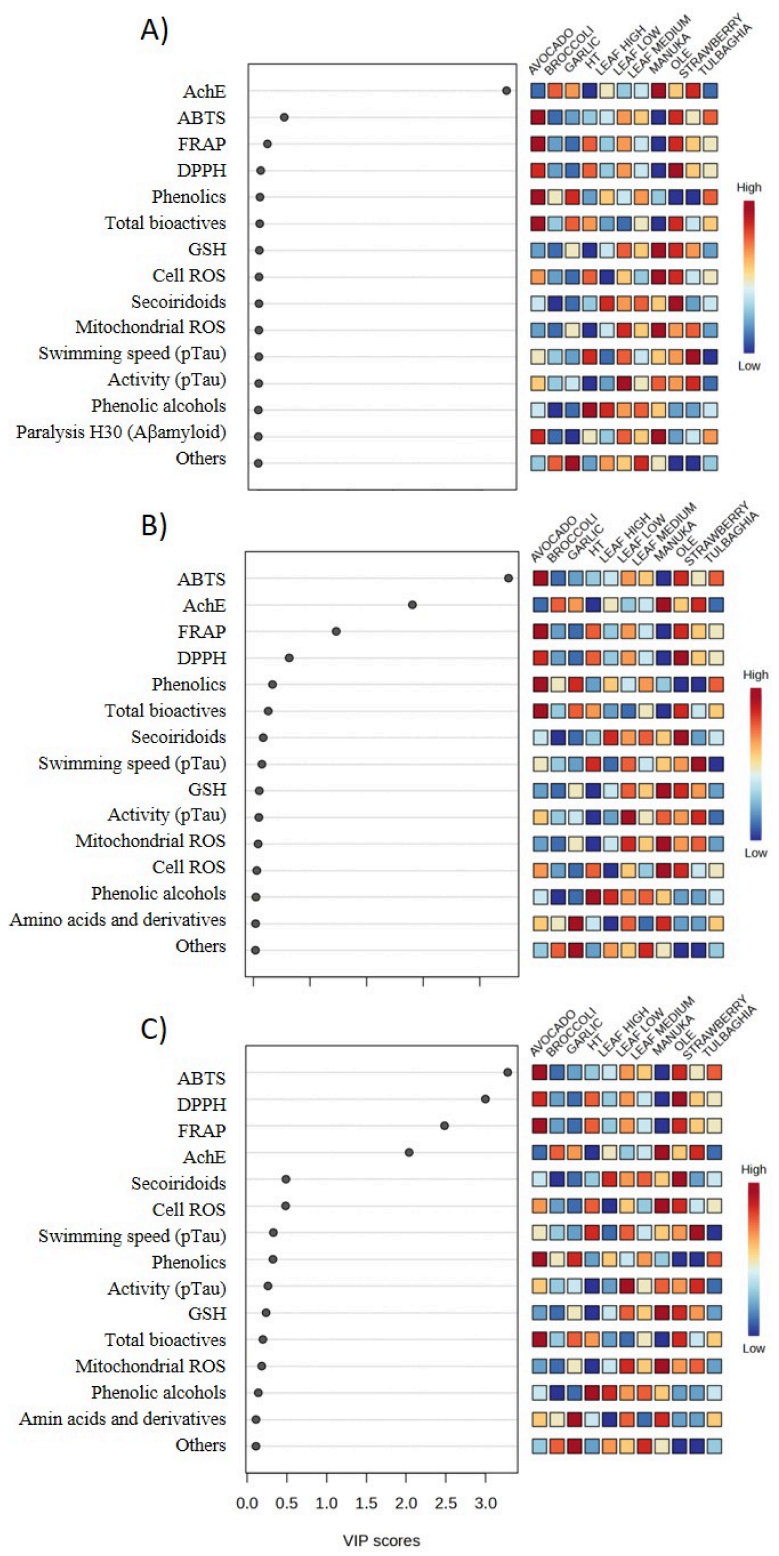
Variable Importance in Projection (VIP) scores from the PLS-DA model: (**A**) Component 1, (**B**) Component 2, and (**C**) Component 3. Each panel displays the ranked contribution of chemical and biological variables to group discrimination, with higher VIP scores indicating greater relevance. The accompanying heatmaps show the relative abundance or response levels of each variable across the eleven analyzed matrices. Abbreviations and original study references: AVOCADO, avocado honey [28]; BROCCOLI, broccoli by-product extract [22]; GARLIC, garlic extract [21]; HT, hydroxytyrosol-rich extract [19]; LEAF HIGH, olive tree leaf extract high in bioactive compounds [20]; LEAF LOW, olive tree leaf extract low in bioactive compounds [20]; LEAF MEDIUM, olive tree leaf extract with medium bioactive compound content [20]; MANUKA, manuka honey [27]; OLE, olive leaf extract rich in oleuropein [18]; STRAWBERRY, strawberry extract [23]; TULBAGHIA, Tulbaghia extract [25]. Other abbreviations: AchE, acetylcholine esterase; ABTS, 2,2′-azinobis(3-ethylbenzothiazoline-6-sulfonic acid); DPPH, 1,1-diphenyl-2-picrylhydrazyl; FRAP, ferric reducing antioxidant power; GSH, reduced Glutathione; pTau, phosphorylated tau protein; ROS, reactive oxygen species.

To identify the most influential variables contributing to class separation, VIP scores were calculated for the first three components of the PLS-DA model (Figure 3). The first component explained 99.6% of the total variance, the second 0.4%, and the third accounted for negligible variance (0%). This high proportion of variance explained by PC1 (99.6%) likely reflects the coexistence of variables spanning different scales and biological domains, rather than a single discriminant biological signal; it should therefore be interpreted with caution as a descriptive summary of the harmonized dataset rather than evidence of a dominant mechanistic axis. However, all three components were retained in the visualization to provide a complete overview of the variable weighting and to ensure the transparency of the multivariate interpretation. In the context of PLS-DA, component variance reflects the proportion of the total variability captured in the predictor space, whereas each VIP component highlights the relative contribution of variables to class discrimination. Thus, even components with minimal variance may refine the weighting of correlated variables without altering the overall pattern of discrimination. Component 1 (Figure 3A) identified acetylcholinesterase inhibitory activity (AChE IC_50_) as the variable with the highest discriminant power (VIP > 5), followed by antioxidant parameters such as ABTS, FRAP, and DPPH radical scavenging capacities and by total phenolic content. These variables collectively drove the separation along the first latent axis, distinguishing highly active matrices (olive-derived extracts and hydroxytyrosol) from less phenolic-rich products (honey and fruit-based samples). Other relevant contributors included total bioactivity and mitochondrial protection (Mitotracker assay), both of which paralleled phenolic concentration. Altogether, Component 1 reflects a general axis of neuroprotective potential and antioxidant strength across the analyzed food-derived compounds. Component 2 (Figure 3B) refined the discrimination by highlighting complementary antioxidant responses. Here, ABTS, AChE IC_50_, and FRAP again ranked among the top variables, but the weighting shifted towards parameters associated with functional bioactivity in vivo, such as tau-related motor assays (TauSwimming and TauActivity) and stress protection in *C. elegans* (MCB N2 and DCFDA (Dichlorodihydrofluorescein diacetate) assays). This secondary axis captures intra-group variability, particularly among olive extracts of different enrichment levels and between honey types, suggesting that distinct extract compositions modulate biological activity beyond total phenolic load. Component 3 (Figure 3C), despite accounting for 0% of the model variance, contributed minor adjustments in variable weighting and was retained for completeness. Its VIP pattern reinforced the dominance of antioxidant indices (ABTS, DPPH, and FRAP) and secondary markers such as AChE inhibition and secoiridoid content, indicating that no new discriminant direction emerged beyond those described in the first two components. Taken together, the VIP analysis across the three latent components confirms that neuroprotective, antioxidant, and phenolic variables constitute the main chemical and functional drivers of class separation. In particular, the consistent prominence of AChE inhibition, phenolic concentration, and redox-related assays underlines the shared biochemical axis through which food-derived compounds may contribute to protection against Alzheimer’s-related dysfunction in the *C. elegans* model.

### 2.3. Correlation Network Between Chemical and Functional Variables

**Figure 4:**
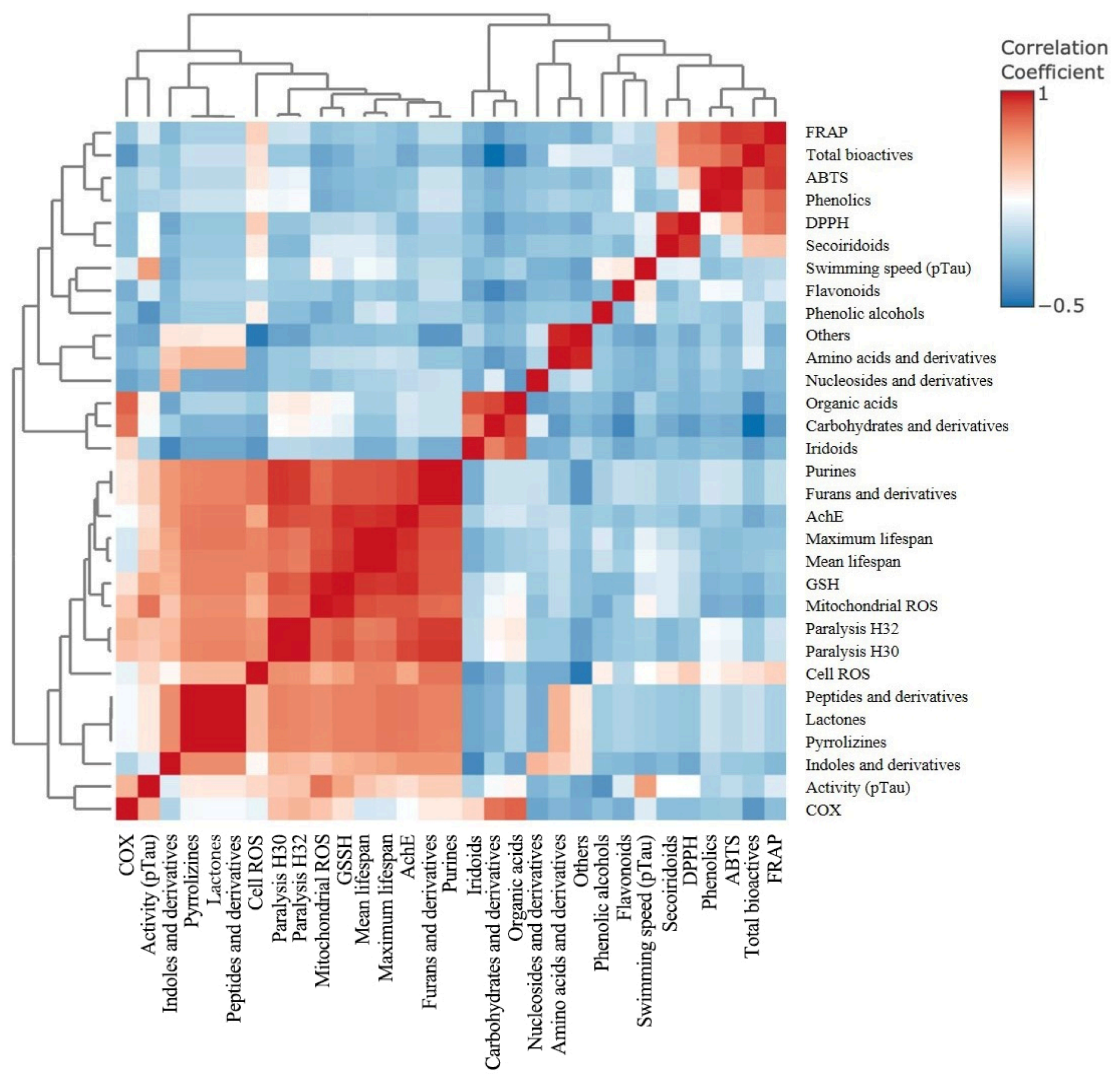
Correlation heatmap of chemical and biological variables across all studied matrices. The color scale represents Pearson correlation coefficients, ranging from −0.5 (blue, negative correlation) to +1.0 (red, positive correlation). The diagonal red band reflects self-correlation of variables, while clustering patterns reveal groups of positively associated chemical classes (e.g., phenolics, iridoids, and flavonoids) and their covariation with biological markers of oxidative stress resistance and neuroprotection. Abbreviations: ABTS, 2,2′-azinobis(3-ethylbenzothiazoline-6-sulfonic acid); ACHE, acetylcholine esterase; COX, cyclooxygenase; DPPH, 1,1-diphenyl-2-picrylhydrazyl; FRAP, ferric reducing antioxidant power; GSH, reduced glutathione; H30, hour 30; H32, hour 32; ROS, reactive oxygen species.

To investigate the relationships between chemical composition and biological performance across the eleven studied matrices, a global correlation analysis was performed using Pearson coefficients (Figure 4). The resulting correlation map revealed two major blocks of positively correlated variables, reflecting the strong coupling between antioxidant composition and neuroprotective outcomes. The first block, positioned in the upper portion of the heatmap, grouped FRAP, ABTS, DPPH, total bioactivity, total phenolics, and secoiridoids, together with Tau Swimming and phenolic alcohols. These variables displayed consistently high positive correlations (r > 0.8), indicating that matrices richer in phenolic and secoiridoid compounds also exhibited stronger antioxidant capacity and improved performance in tauopathy-related assays. This cluster reflects the chemical and functional signature of the olive-derived extracts, characterized by potent redox modulation and neuromotor protection in *C. elegans*. In contrast, a second cluster located in the lower-right quadrant included oxidative stress and functional vulnerability markers such as DCFDA protection, MCB N2 (% stressed), and ParalysisH30/H32, together with lifespan-related parameters. These variables were negatively correlated with antioxidant and phenolic indices, confirming that extracts with higher antioxidant potency were associated with reduced oxidative burden and lower amyloid-induced paralysis. Intermediate correlations were observed for AChE IC_50_ and mitochondrial protection (Mitotracker), which bridged the chemical and biological dimensions, linking phenolic-driven antioxidant mechanisms with cholinergic modulation and preservation of mitochondrial function. The observed structure highlights an integrated network of associations connecting redox-active phenolics, secoiridoids, and flavonoids with downstream functional protection against oxidative and proteotoxic stress. Overall, the correlation map provides a comprehensive overview of how the chemical architecture of the tested food-derived compounds underpins their biological effects. The close alignment of compositional and functional markers supports a mechanistic continuum linking antioxidant defense, protein aggregation control, and neuronal function preservation in *C. elegans* models of Alzheimer’s-related pathology.

### 2.4. Hierarchical Clustering of Bioactive Chemotypes

**Figure 5:**
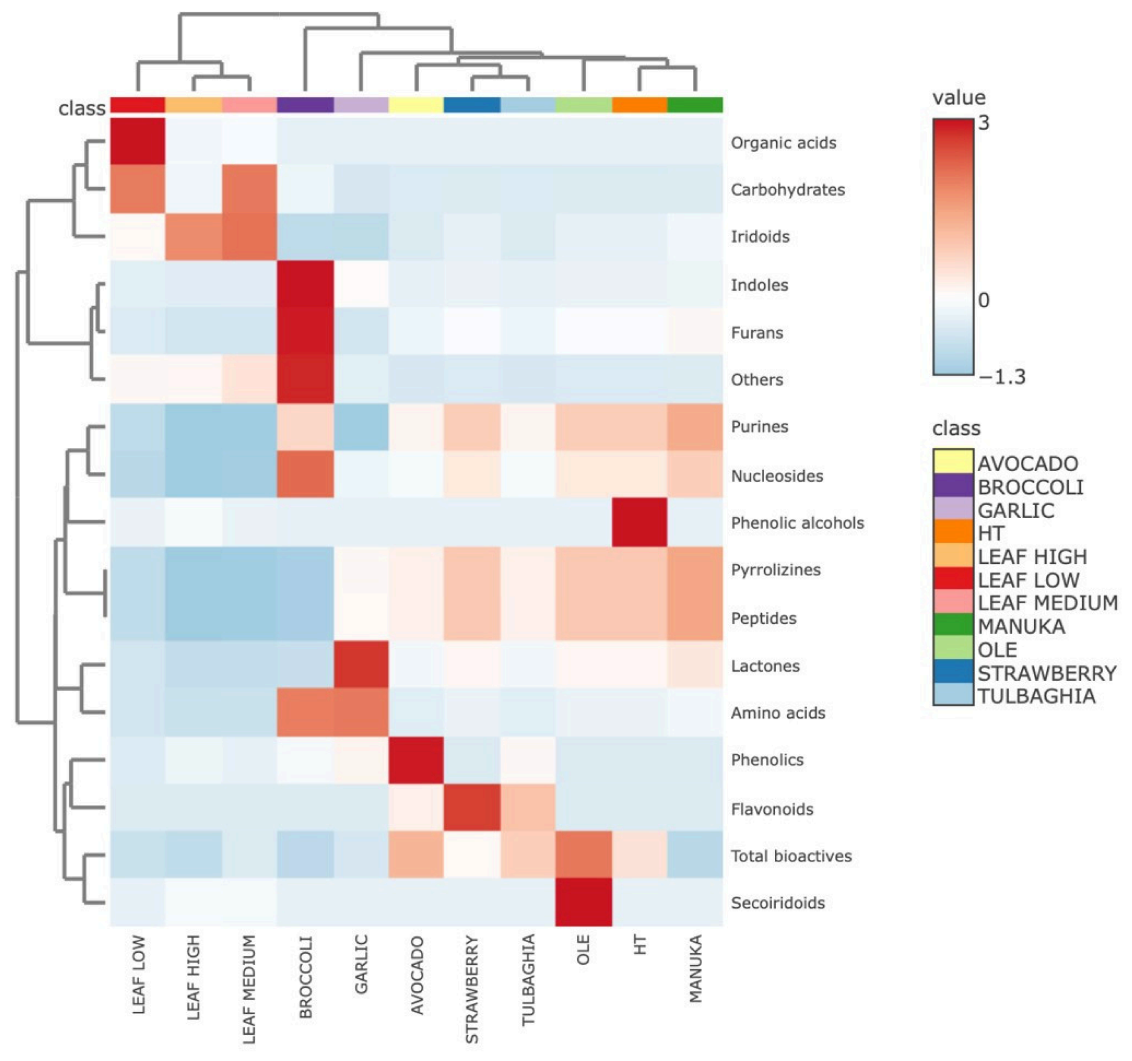
Hierarchical clustering heatmap of metabolite classes across all tested matrices. The color scale represents normalized abundance values (blue: low; red: high). Dendrograms depict similarity relationships between samples and compound categories, revealing distinct chemical profiles that group together matrices sharing predominant metabolite families. Extracts enriched in phenolic alcohols, flavonoids, or secoiridoids clustered apart from those dominated by peptides, purines, or pyrrolizine derivatives. Abbreviations and original study references: AVOCADO, avocado honey [28]; BROCCOLI, broccoli by-product extract [22]; GARLIC, garlic extract [21]; HT, hydroxytyrosol-rich extract [19]; LEAF HIGH, olive tree leaf extract high in bioactive compounds [20]; LEAF LOW, olive tree leaf extract low in bioactive compounds [20]; LEAF MEDIUM, olive tree leaf extract with medium bioactive compound content [20]; MANUKA, manuka honey [27]; OLE, olive leaf extract rich in oleuropein [18]; STRAWBERRY, strawberry extract [23]; TULBAGHIA, Tulbaghia extract [25].

To further explore how the compositional profiles of the eleven matrices related to each other, a two-dimensional hierarchical clustering analysis (HCA) was performed based on normalized metabolomic features (Figure 5). This approach allowed simultaneous clustering of both variables (chemical classes) and samples (matrices), revealing distinct compositional patterns associated with botanical origin. Three main clusters of samples were identified. The first group comprised olive-derived matrices—including hydroxytyrosol (HT), olive leaf extracts at different enrichment levels (LEAF LOW, MEDIUM, and HIGH), and OLE—characterized by high levels of secoiridoids, phenolics, and phenolic alcohols, together with elevated total bioactivity. This tight cluster reflects the chemical homogeneity and shared metabolic backbone of these extracts, consistent with the results of PCA, k-means clustering, and PLS-DA analyses. The second cluster grouped garlic and broccoli, which showed enrichment in organic acids, carbohydrates, and iridoid-related compounds and a distinct deficiency in phenolic and secoiridoid markers. These features are typical of matrices rich in sulfur- and glucosinolate-derived metabolites, as expected for members of the Allium and Brassica genera. The third cluster included honey and fruit-derived products (avocado, manuka, and strawberry), which were associated with moderate levels of flavonoids, purines, and nucleosides, as well as selected amino acid derivatives and lactones. This cluster reflects a metabolomic profile driven by nectar- and fruit-based constituents rather than phenolic secoiridoids. Interestingly, *Tulbaghia* showed partial overlap with this group, consistent with its mixed profile containing both sulfur-related and phenolic metabolites. The variable dendrogram highlighted two major compositional domains: one encompassing phenolic and redox-active compounds (secoiridoids, flavonoids, phenolic alcohols, and total bioactives) and another defined by primary metabolites (carbohydrates, amino acids, nucleosides, and organic acids). The organization of these clusters underscores the chemical logic behind the biological classification observed in supervised analyses: samples that share phenolic-driven metabolomic signatures also exhibit stronger antioxidant and neuroprotective outcomes. Together with the correlation matrix, the HCA confirms the internal coherence of the integrated dataset and reinforces the concept of a functional chemotype linking the phytochemical composition of food-derived compounds with their biological efficacy in neurodegenerative models.

### 2.5. Connecting Chemical Profiles to Functional Outcomes

#### 2.5.1. Oxidative Stress Protection (DCFDA Assay)

**Figure 6:**
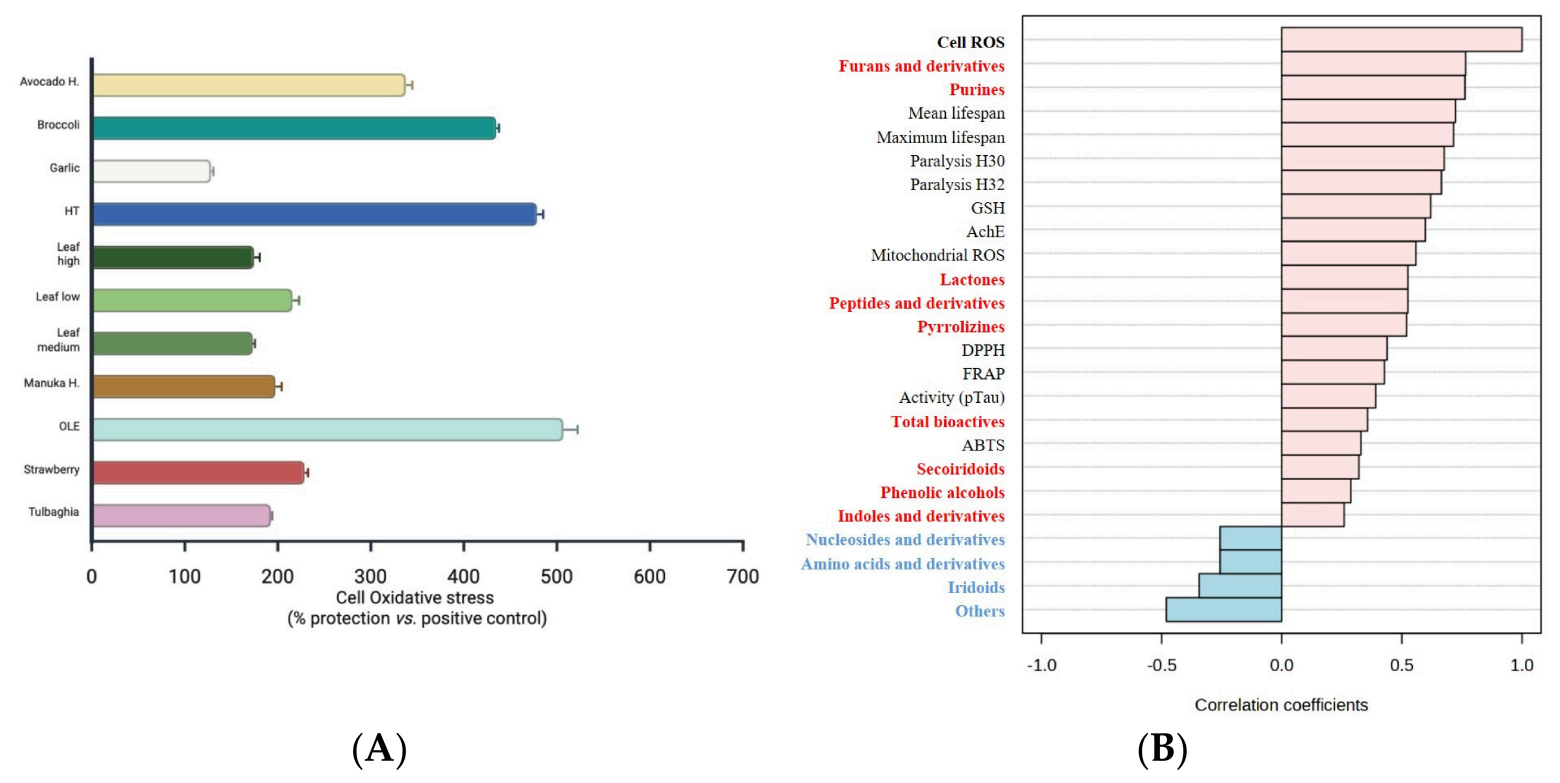
Correlation of chemical composition with oxidative stress protection (DCFDA assay). (**A**) Comparative oxidative stress protection (% protection vs. positive control) across all tested matrices. (**B**) Correlation coefficients between DCFDA-derived antioxidant protection and chemical or biological variables. Positive correlations (red) indicate compound classes or endpoints associated with higher oxidative defense, whereas negative correlations (blue) denote inverse relationships. Red and blue colors highlight metabolite families identified in the chemical composition of the tested extracts, illustrating their contribution to functional outcomes. Abbreviations and original study references: Avocado H., avocado honey [28]; Broccoli, broccoli by-product extract [22]; Garlic, garlic extract [21]; HT, hydroxytyrosol-rich extract [19]; Leaf high, olive tree leaf extract high in bioactive compounds [14]; Leaf low, olive tree leaf extract low in bioactive compounds [20]; Leaf medium, olive tree leaf extract with medium bioactive compound content [20]; Manuka H., manuka honey [27]; OLE, olive leaf extract rich in oleuropein [18]; Strawberry, strawberry extract [23]; Tulbaghia, Tulbaghia extract [25]. Other abbreviations: ABTS, 2,2′-azinobis(3-ethylbenzothiazoline-6-sulfonic acid); AChE, acetylcholine esterase; DPPH, 1,1-diphenyl-2-picrylhydrazyl; FRAP, ferric reducing antioxidant power; GSH, reduced glutathione; pTau, phosphorylated tau protein; ROS, reactive oxygen species.

To dissect how chemical composition translated into oxidative stress protection, correlation analyses were conducted using DCFDA as the reference variable (Figure 6). The bar plot on the left illustrates the percentage of protection against induced oxidative stress across the eleven matrices, while the correlation chart on the right identifies the biochemical and functional variables most strongly associated with DCFDA protection. It should be noted that DCFDA is a general readout of intracellular oxidative burden and is not mitochondria-specific; therefore, these results should not be interpreted as direct evidence of mitochondrial protection or mitophagy.

Olive-derived matrices, particularly hydroxytyrosol (HT) and olive leaf extract (OLE), exhibited the highest levels of cellular protection, achieving reductions of up to 600% compared with positive controls. Intermediate protection was observed for broccoli and high-enrichment leaf extracts, whereas garlic and *Tulbaghia* showed limited efficacy, consistent with their lower phenolic content. The correlation analysis revealed that DCFDA protection was positively associated with several compositional and bioactivity markers, including furans and derivatives, purines, lactones, peptides and derivatives, pyrrolizines, and total bioactivity, as well as antioxidant parameters (FRAP, DPPH, and ABTS). These associations underscore the contribution of both phenolic and secondary metabolites of distinct chemical origin to redox homeostasis. Conversely, phenolic alcohols, indole derivatives, nucleosides, amino acids, and iridoids displayed negative correlations, reflecting their relative enrichment in matrices with weaker antioxidant protection. This inverse relationship suggests that not all phenolic-related compounds contribute equally to oxidative defense and that specific subclasses—particularly secoiridoids and conjugated furans—play a more determinant role in ROS modulation.

Overall, this analysis demonstrates that oxidative stress protection in *C. elegans* is driven by a combination of phenolic-rich and furan–pyrrolizine-linked metabolites, highlighting the unique chemical fingerprint of olive-derived products as the most effective antioxidant bioactive compounds within the dataset.

#### 2.5.2. β-Amyloid Paralysis Assay (32 h)

**Figure 7:**
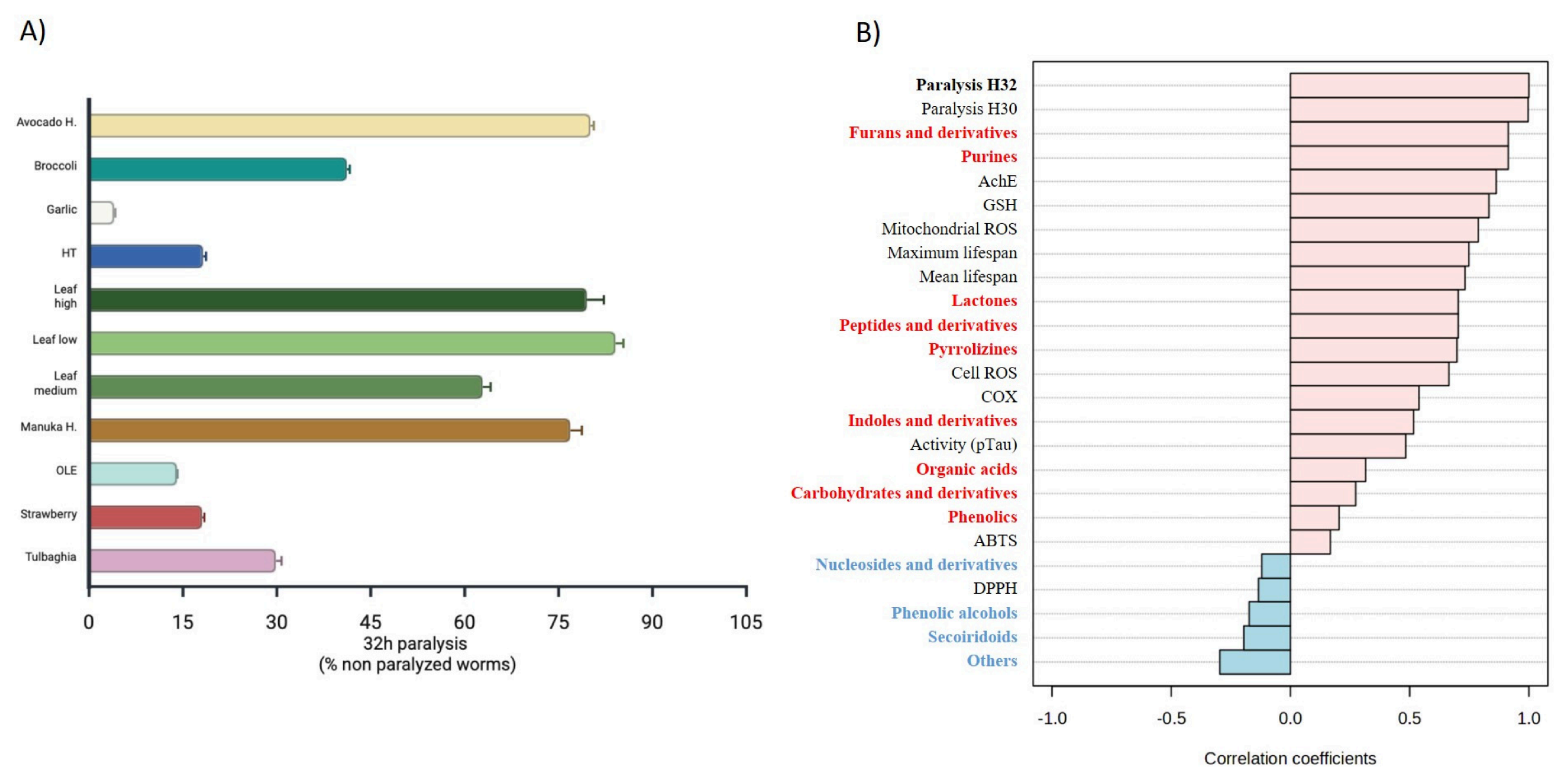
Correlation of chemical composition with β-amyloid proteotoxicity protection (paralysis at 32 h). (**A**) Comparative percentage of non-paralyzed *C. elegans* after 32 h exposure to β-amyloid, showing differential protection across tested matrices. (**B**) Correlation coefficients between the proportion of non-paralyzed worms and chemical or biological variables. Positive correlations (red) indicate metabolite classes or functional endpoints associated with enhanced protection against amyloid-induced paralysis, whereas negative correlations (blue) reflect inverse associations. Red and blue colors highlight compound families identified in the chemical composition of the analyzed extracts. Abbreviations and original study references: Avocado H., avocado honey [28]; Broccoli, broccoli by-product extract [22]; Garlic, garlic extract [21]; HT, hydroxytyrosol-rich extract [19]; Leaf high, olive tree leaf extract high in bioactive compounds [20]; Leaf low, olive tree leaf extract low in bioactive compounds [20]; Leaf medium, olive tree leaf extract with medium bioactive compound content [20]; Manuka H., manuka honey [27]; OLE, olive leaf extract rich in oleuropein [18]; Strawberry, strawberry extract [23]; Tulbaghia, Tulbaghia extract [25]. Other abbreviations: ABTS, 2,2′-azinobis(3-ethylbenzothiazoline-6-sulfonic acid); AChE, acetylcholine esterase; DPPH, 1,1-diphenyl-2-picrylhydrazyl; FRAP, ferric reducing antioxidant power; GSH, reduced glutathione; pTau, phosphorylated tau protein; ROS, reactive oxygen species.

The β-amyloid-induced paralysis assay provided a functional readout of neuroprotective capacity in the transgenic *C. elegans* CL4176 strain, which expresses human Aβ_1−42_ in body wall muscle cells. The percentage of non-paralyzed worms at 32 h after induction reflects the ability of each matrix to counteract amyloid toxicity (Figure 7).

Olive leaf extracts—particularly those with medium and high enrichment levels—showed the strongest protective effects, with over 70–80% of worms remaining motile after 32 h. These extracts were followed by manuka honey and hydroxytyrosol, which maintained intermediate levels of protection, while strawberry, garlic, and *Tulbaghia* exhibited limited efficacy. This pattern parallels, although not perfectly, the antioxidant trend observed in the DCFDA assay, suggesting both overlapping and distinct mechanistic contributions. The correlation analysis revealed a positive association of anti-paralysis activity with chemical variables such as furans and derivatives, purines, lactones, peptides and derivatives, and pyrrolizines, indicating that these structural classes may contribute to the modulation of amyloid aggregation or related proteotoxic stress responses. Additionally, variables linked to organic acids, carbohydrates, and indole derivatives also correlated positively, pointing to a potential synergistic contribution of redox-active and metabolic intermediates. Conversely, secoiridoids, phenolic alcohols, and nucleosides displayed negative correlations with the anti-paralytic phenotype. This finding suggests that, although olive-derived compounds are functionally effective, their activity may rely more on derived or co-extracted metabolites (e.g., conjugated lactones and pyrrolizines) than on the canonical phenolic backbone itself. Taken together, these results reveal a composite neuroprotective signature where both phenolic and non-phenolic compounds participate in attenuating β-amyloid toxicity. The high performance of olive leaf matrices within this network reinforces their multifactorial potential as modulators of amyloidogenic processes, extending beyond direct antioxidant action.

#### 2.5.3. Tauopathy-Related Motility (Swimming Assay)

**Figure 8:**
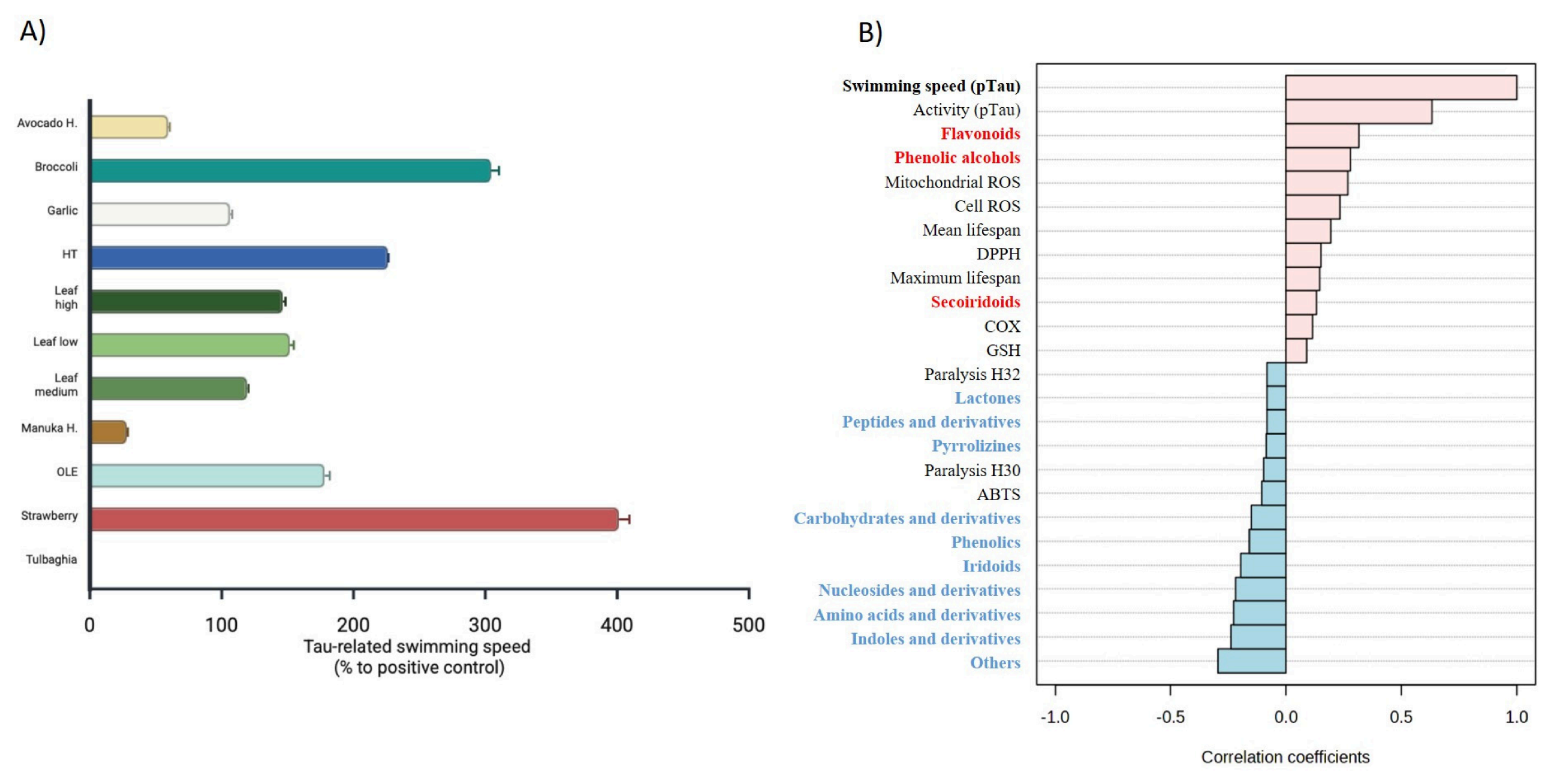
Correlation of chemical composition with tau-associated neuroprotection (Tau-swimming assay). (**A**) Comparative swimming velocity of *C. elegans* expressing phosphorylated tau, expressed as percentage relative to the positive control. (**B**) Correlation coefficients between Tau-swimming performance and chemical or biological variables. Positive correlations (red) denote metabolite families or functional parameters associated with improved motility and protection against tau-induced toxicity, whereas negative correlations (blue) indicate adverse or unrelated associations. Red and blue colors highlight compound classes present in the chemical profiles of the tested matrices. Abbreviations and original study references: Avocado H., avocado honey [28]; Broccoli, broccoli by-product extract [22]; Garlic, garlic extract [21]; HT, hydroxytyrosol-rich extract [19]; Leaf high, olive tree leaf extract high in bioactive compounds [20]; Leaf low, olive tree leaf extract low in bioactive compounds [20]; Leaf medium, olive tree leaf extract with medium bioactive compound content [20]; Manuka H., manuka honey [27]; OLE, olive leaf extract rich in oleuropein [18]; Strawberry, strawberry extract [23]; Tulbaghia, Tulbaghia extract [25]. Other abbreviations: ABTS, 2,2′-azinobis(3-ethylbenzothiazoline-6-sulfonic acid); COX, cyclooxygenase; DPPH, 1,1-diphenyl-2-picrylhydrazyl; GSH, reduced glutathione; H30, hour 30; H32, hour 32; ROS, reactive oxygen species.

The swimming assay in the *C. elegans* tauopathy model provided an additional functional readout of neurodegenerative impairment associated with tau hyperphosphorylation. In this strain, the reduction in body movement speed reflects neuronal and muscular dysfunction triggered by tau aggregation. The percentage of swimming speed relative to positive control animals quantifies the capacity of each matrix to mitigate tau-induced motility loss (Figure 8). A distinctive pattern emerged compared with amyloid-related assays. Strawberry extract exhibited the strongest protective effect, restoring motility to nearly 400% of the control level, followed by broccoli, hydroxytyrosol (HT), and olive leaf extracts, which also significantly improved swimming performance. Conversely, garlic, *Tulbaghia*, and manuka honey showed negligible activity. Correlation analysis identified flavonoids, phenolic alcohols, and secoiridoids as positively associated with motility preservation, supporting their role in modulating tau-related dysfunctions. These results suggest that polyphenols, especially flavonoids and tyrosol/oleuropein derivatives, contribute to neuronal resilience, possibly through mechanisms involving protein homeostasis or anti-inflammatory modulation beyond their direct antioxidant effects. In contrast, lactones, peptides and derivatives, and pyrrolizines showed negative correlations, together with broad categories such as carbohydrates, nucleosides, and amino acid derivatives, indicating that matrices enriched in these compounds (e.g., garlic and manuka) are less effective against tau-induced motility decline. Overall, the tauopathy assay revealed a more selective contribution of phenolic subclasses compared to β-amyloid or oxidative stress models. The strong association of motility recovery with flavonoids and phenolic alcohols reinforces their mechanistic relevance in targeting tau pathology and maintaining neuronal function in *C. elegans*.

#### 2.5.4. Cholinergic Modulation (AChE Inhibition)

**Figure 9:**
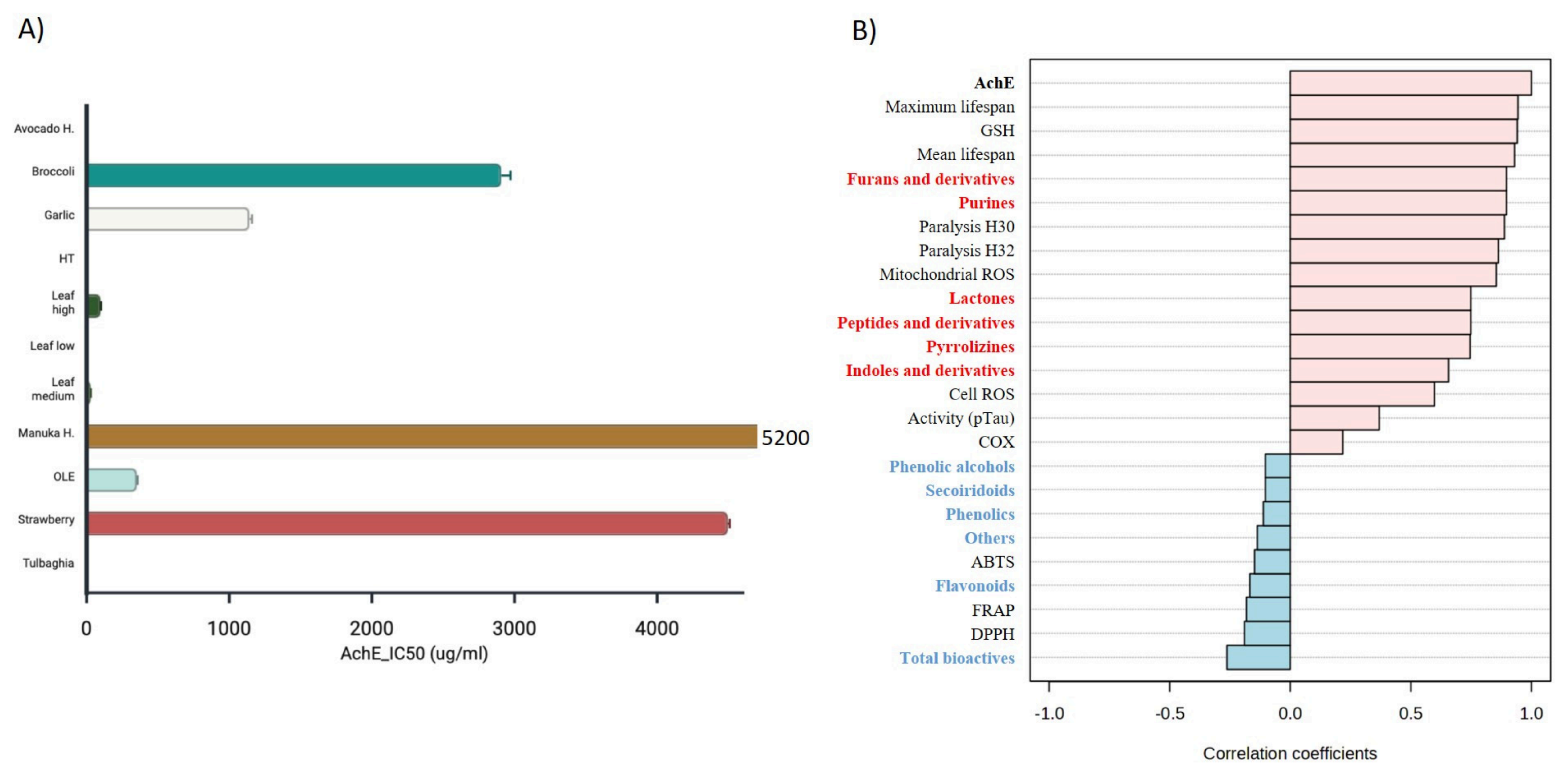
Correlation of chemical composition with acetylcholinesterase inhibitory activity (AChE IC_50_). (**A**) Comparative IC_50_ values for acetylcholinesterase inhibition across all tested matrices. Lower values indicate stronger inhibitory potency. (**B**) Correlation coefficients between AChE inhibitory activity and chemical or biological variables. Positive correlations (red) correspond to metabolite families or functional endpoints associated with enhanced cholinesterase inhibition, whereas negative correlations (blue) indicate inverse or non-contributory relationships. Red and blue colors highlight compound families identified in the chemical composition of the evaluated extracts. Abbreviations and original study references: Avocado H., avocado honey [28]; Broccoli, broccoli by-product extract [22]; Garlic, garlic extract [21]; HT, hydroxytyrosol-rich extract [19]; Leaf high, olive tree leaf extract high in bioactive compounds [20]; Leaf low, olive tree leaf extract low in bioactive compounds [20]; Leaf medium, olive tree leaf extract with medium bioactive compound content [20]; Manuka H., manuka honey [27]; OLE, olive leaf extract rich in oleuropein [18]; Strawberry, strawberry extract [23]; Tulbaghia, Tulbaghia extract [25]. Other abbreviations: ABTS, 2,2′-azinobis(3-ethylbenzothiazoline-6-sulfonic acid); COX, cyclooxygenase; DPPH, 1,1-diphenyl-2-picrylhydrazyl; FRAP, ferric reducing antioxidant power; GSH, reduced glutathione; H30, hour 30; H32, hour 32; pTau, phosphorylated tau protein; ROS, reactive oxygen species.

Acetylcholinesterase (AChE) inhibition represents a key therapeutic strategy in Alzheimer’s disease aimed at maintaining acetylcholine availability in synaptic clefts. Although this assay was not performed for all matrices in the original studies, available data for a representative subset were integrated to preserve the neurofunctional dimension of the analysis (Figure 9). Among the tested matrices, broccoli and manuka honey displayed the highest AChE inhibitory activity (lower IC_50_ values), followed by garlic and olive leaf extract, whereas strawberry and avocado honey exhibited comparatively weaker effects. This partial overlap with oxidative and amyloid assays highlights the complexity of biochemical determinants underlying cholinergic modulation. The correlation analysis indicated positive associations of AChE inhibition with furans and derivatives, purines, lactones, peptides, and indole-related compounds, suggesting that nitrogen-containing and heterocyclic structures may contribute to cholinesterase inhibition. Conversely, phenolic alcohols, secoiridoids, and flavonoids were negatively correlated, possibly reflecting that matrices rich in canonical phenolics (such as olive-derived extracts) act primarily through antioxidant or anti-aggregation mechanisms rather than direct cholinergic modulation. Despite its limited dataset, the inclusion of AChE activity provides a valuable complementary view linking antioxidant, anti-amyloid, and anti-tau effects to cholinergic pathways—reinforcing the multifactorial neuroprotective profile of food-derived compounds explored in this study.

### 2.6. Overall Integration and Cross-Marker Relationships

When the datasets were harmonized and jointly analyzed using MetaboAnalyst, multivariate models revealed consistent clustering patterns linking specific metabolite families with neuroprotective activity. Principal Component Analysis (PCA) and k-means clustering confirmed clear separation between extracts with demonstrated bioactivity and those with limited effects, suggesting shared chemical signatures across matrices of different origin. Partial Least Squares Discriminant Analysis (PLS-DA) further highlighted phenolic alcohols, flavonoids, secoiridoids, and sulfur-containing compounds as major contributors to group discrimination, as confirmed by high Variable Importance in Projection (VIP) scores. Hierarchical clustering and correlation analyses reinforced these associations, revealing co-occurrence networks among polyphenols, iridoids, and biological endpoints such as oxidative stress resistance and reduced β-amyloid toxicity. Altogether, these results support the existence of a common metabolomic fingerprint underlying the neuroprotective effects of diverse food-derived compounds, providing a unified biochemical perspective that could guide rational formulation strategies combining complementary compound families with proven bioactivity.

## 3. Discussion

The integrated metabolomic analysis revealed consistent chemical and biological patterns across food-derived compounds with demonstrated neuroprotective activity. Despite their diverse botanical and compositional origins, matrices such as olive by-products, strawberry, broccoli, garlic, honey, and edible flowers shared overlapping signatures associated with oxidative stress resistance, improved proteostasis, and cholinergic modulation. These findings suggest the existence of a convergent biochemical framework underlying the neuroprotective effects of plant- and food-derived products, where phenolic and non-phenolic compounds act synergistically to modulate key pathways implicated in Alzheimer’s disease.

Although the present re-analysis was limited to studies conducted within our own laboratory, this focused approach should be regarded as a methodological strength rather than a limitation [31]. All experiments were carried out in the same laboratory, under rigorously standardized conditions, using identical *Caenorhabditis elegans* models, exposure protocols, and analytical platforms. This high degree of internal consistency minimizes confounding variability and allows consistent cross-comparison of both chemical and biological data. Such uniformity is rarely achievable in conventional meta-analyses combining heterogeneous studies from multiple laboratories, where methodological differences often obscure mechanistic interpretation. In this context, the restricted yet homogeneous dataset enhances the interpretability and internal coherence of the metabolomic patterns identified, providing exploratory insights into the shared biochemical signatures underlying neuroprotective activity.

### 3.1. Shared Biochemical Mechanisms and Matrix-Dependent Differences

The integrative metabolomic approach revealed that the neuroprotective activity observed across matrices does not rely on a single class of bioactive compounds, but rather on the complementary contribution of multiple chemical families. Phenolic alcohols, secoiridoids, and flavonoids consistently emerged as major positive correlates of functional protection in assays addressing oxidative stress, β-amyloid toxicity, and tau-related motility. These compound classes are known to modulate redox balance, mitochondrial function, and proteostasis, mechanisms that are all central to Alzheimer’s disease pathology [32,33,34,35].

Given the central role of mitochondrial dysfunction in Alzheimer’s disease, it is worth considering the observed redox protection in relation to mitochondrial quality control [36,37]. Hence, Castellazzi et al. reported reduced circulating levels of ATG5 and Parkin in patients with AD and mild cognitive impairment, pointing to impaired autophagy/mitophagy pathways in cognitive decline [38]. This provides a plausible, though strictly hypothetical, framework in which the redox protection observed here could be linked to mitochondrial homeostasis [35,39,40]. Future studies should incorporate mitochondria-specific endpoints—mitochondrial ROS, membrane potential, respiratory activity, and mitochondrial morphology—together with mitophagy-flux markers such as *pink-1*, *pdr-1*/Parkin, *dct-1*, and *lgg-1* in *C. elegans* to test this hypothesis directly.

Under the conditions studied, the extracts derived from olive by-products and strawberry shared the most pronounced bioactivity profiles, showing strong and recurrent associations between phenolic subclasses and functional endpoints. Their activity patterns support the notion of a polyphenol-driven metabolic signature that underpins protection against oxidative and proteotoxic stress [16,41,42]. In contrast, matrices enriched in sulfur-containing metabolites—such as garlic and *Tulbaghia*—showed a distinct chemical and functional profile, characterized by moderate antioxidant activity but weaker effects on amyloid and tau models. This suggests that their neuroprotective potential may operate through alternative mechanisms, including modulation of cellular redox signaling and xenobiotic metabolism [43,44].

The analysis also highlighted that honey matrices behaved as functional outliers. Although they possess moderate antioxidant and cholinergic effects, their performance in motility-based assays was markedly reduced. This pattern aligns with previous observations from individual studies, where the high sugar content of honeys was found to adversely affect *C. elegans* motility and energy balance, acting as a confounding variable that may mask the contribution of minor bioactive components [27,28,45]. This emphasizes the importance of considering the matrix context—not only the active molecules—when interpreting biological outcomes [46,47].

Taken together, these results support a model in which different food matrices converge on common neuroprotective pathways through distinct but complementary chemical routes. Polyphenol-rich products primarily target oxidative and proteostatic mechanisms, while sulfur- and indole-containing metabolites may contribute to redox and metabolic adaptation. Such diversity in chemical scaffolds and mechanisms ultimately reinforces the potential of food-derived compounds to act synergistically in multifactorial conditions such as Alzheimer’s disease [48,49,50].

### 3.2. Metabolomic Fingerprint and Translational Relevance

Beyond the identification of individual compound classes, the integrative multivariate analysis revealed a shared metabolomic fingerprint that characterizes the neuroprotective action of diverse food-derived matrices. This fingerprint integrates features from multiple chemical domains—particularly phenolic alcohols, flavonoids, iridoids, and certain sulfur- and nitrogen-containing metabolites—that collectively contribute to redox regulation, maintenance of protein homeostasis, and synaptic function. Such a pattern suggests that food-derived products’ efficacy arises not from isolated molecules, but from combinatorial effects within complex phytochemical environments [51,52]. The observed convergence between chemically distinct matrices, such as olive by-products and strawberry, highlights the existence of conserved metabolic pathways through which different bioactive profiles can lead to similar neurofunctional outcomes [53,54]. This notion aligns with systems-level models of Alzheimer’s disease, where oxidative stress, amyloid aggregation, and tau hyperphosphorylation are interconnected processes amenable to simultaneous modulation by multi-target agents [6]. From a translational perspective, the identification of this common metabolomic signature offers a rational framework for the design of next-generation food-derived formulations. By combining complementary compound families—such as phenolic alcohols and flavonoids with sulfur- or indole-based metabolites—it may be possible to enhance efficacy through synergistic modulation of cellular stress responses, neurotransmission, and proteostatic balance. Moreover, the use of *C. elegans* as an integrative screening model provides a cost-effective, ethically sustainable, and mechanistically relevant platform to prioritize such compound combinations before moving to mammalian systems [55,56,57]. Altogether, the integrated metabolomic re-analysis supports a multi-compound, multi-target paradigm for nutritional neuroprotection, emphasizing that functional outcomes in complex diseases like Alzheimer’s are better explained by network interactions among metabolites than by single-molecule effects.

Building upon the observed compositional and functional diversity among the tested matrices, we explored the potential for rational combinations of food-derived extracts that may maximize coverage across key neuroprotective domains. While direct synergistic interactions remain to be empirically demonstrated, the integrated analysis of antioxidant capacity, enzyme inhibition, and protection against protein aggregation allowed us to identify complementary bioactivity patterns among specific pairs or triplets of extracts. These observations support the idea that combining matrices with distinct but overlapping functional profiles—such as polyphenol-rich olive leaf extracts with fermentable honey derivatives or sulfur-containing compounds from garlic with purine-rich matrices—may broaden the protective scope against Alzheimer-related phenotypes.

### 3.3. Strengths, Limitations, and Perspectives

The present study demonstrates the potential of integrative metabolomic approaches to extract new mechanistic insights from existing experimental datasets. By combining harmonized data from nine independent studies under a unified analytical framework, the work establishes a reproducible strategy to evaluate the chemical–biological coherence of the observed bioactive effects. This approach contributes to the field of nutritional neuroscience by bridging compositional chemistry and functional bioassays through systems-level analysis. A major strength of the study lies in the high methodological consistency across all included datasets, which ensures that observed differences arise from matrix composition rather than from experimental variability. The focused scope—restricted to internally generated studies—allowed reliable cross-comparison of neuroprotective patterns, minimizing confounding factors and improving interpretability [31]. The resulting homogeneity contrasts with conventional meta-analyses that often integrate heterogeneous protocols, analytical methods, or biological models [58,59]. Nonetheless, certain limitations must be acknowledged. The use of summarized data rather than raw replicates may reduce statistical power and limit the precision of quantitative inferences [60]. Consequently, PCA, k-means clustering, PLS-DA, VIP scores, and correlation analyses reported throughout this study should be explicitly regarded as exploratory and hypothesis-generating tools rather than validated discriminant or predictive models. No permutation testing, cross-validation, or other inferential procedures were performed, given the summarized nature of the source data. Similarly, while the *C. elegans* model provides a robust platform to probe conserved neuroprotective mechanisms, extrapolation to the human context requires further validation [61,62]. Future work should expand the analysis to external datasets and complementary experimental systems to test the generalizability of the identified metabolomic patterns. Despite these constraints, the study offers a proof of concept for the application of data integration and metabolomic modeling to food-derived applications research. The identification of a reproducible chemical–functional profile opens the way toward evidence-based formulation strategies, combining complementary compound families for enhanced neuroprotection. In this context, the use of the term metabolomic fingerprint refers to the descriptive integration of co-occurring chemical and functional features, rather than to a validated predictive model—thereby underscoring the exploratory and hypothesis-generating nature of the present work. Additionally, the integrative metabolomic analysis allowed the generation of rationale-based hypotheses regarding potential combinations of food-derived extracts with complementary bioactivities. Although these proposals remain speculative and should be interpreted with caution, they open interesting avenues for future experimental validation. Such follow-up studies could explore whether mixtures based on the present compositional and functional data may offer additive or synergistic effects in the context of neuroprotection and healthy aging.

## 4. Materials and Methods

### 4.1. Data Sources and Scope

This study was based exclusively on data extracted from previously published research articles authored by our group between 2021 and 2025 [18,19,20,21,22,23,25,27,28]. Table 1 summarizes the origin of food-derived matrices employed for the data. All the included studies investigated food-derived products or by-products as sources of bioactive compounds with neuroprotective potential, using *Caenorhabditis elegans* as an in vivo experimental model. Each study evaluated comparable biological endpoints—such as oxidative stress resistance, β-amyloid-induced proteotoxicity, and lifespan extension—and provided metabolomic or chemical characterization of the tested extracts. The chemical profiles were originally obtained by liquid chromatography coupled to mass spectrometry. All datasets and related analytical details are publicly available in the corresponding publications. It should be noted that the integrated dataset was deliberately restricted to studies conducted within our own laboratory. While this approach necessarily limits the diversity of matrices represented, it ensures full methodological consistency across experiments, including identical *C. elegans* strain culture and handling, experimental conditions, and analytical workflows. This homogeneity strengthens the reliability of cross-study comparisons and the validity of the metabolomic integration performed [63,64].

### 4.2. Data Extraction and Harmonization

Quantitative data were extracted directly from tables and figures available in the published papers (Table 1). Only variables for which numerical information could be reliably obtained were included; no new experimental work or unpublished data were generated. Data were curated to ensure consistency in variable nomenclature, units, and experimental context (strain background, exposure period, and assay endpoints). This harmonization step allowed cross-comparison among the nine studies, all of which had been conducted under analogous methodological frameworks.

### 4.3. Data Formatting and Synthetic Replication

Because MetaboAnalyst requires a minimum of three replicates per group to perform multivariate analyses, synthetic triplicates were generated from the mean  ±  standard error values reported in the original publications [29]. For each variable, three data points were constructed corresponding to (i) the reported mean, (ii) the mean plus the standard error, and (iii) the mean minus the standard error. This procedure enabled statistical processing of datasets originally available as summarized values only, while preserving the relative variability and scale of the published data. The resulting synthetic matrices were used solely for exploratory and comparative analyses; no inferential statistics were drawn from these simulated replicates.

### 4.4. Metabolomic Data Integration and Analysis

The harmonized datasets were reprocessed using the MetaboAnalyst 6.0 platform (https://www.metaboanalyst.ca, accessed on 29 June 2026) for multivariate and univariate analysis [29]. Each variable (representing identified metabolites or compound features) was log-transformed and auto-scaled prior to analysis. Principal Component Analysis (PCA), Partial Least Squares Discriminant Analysis (PLS-DA), Variable Importance in Projection (VIP) scoring, k-means clustering, and hierarchical clustering were performed to identify patterns of covariance and compound families consistently associated with neuroprotective outcomes [65]. These analyses were applied to the compiled chemical and biological datasets to reveal convergent metabolomic profiles across the nine independent studies.

Given that the dataset combined variables of markedly different biological natures and numerical scales (e.g., IC_50_ values in µg/mL, percentage-based bioassay readouts, and relative metabolite abundances), auto-scaling and log-transformation were applied specifically to prevent any single variable class from disproportionately dominating the multivariate models. Nonetheless, the possibility that endpoints with comparatively larger dynamic ranges influenced the separation observed along PC1 cannot be fully excluded.

### 4.5. Statistical Analysis

All data analyses were conducted using the statistical modules implemented in MetaboAnalyst 6.0 [29]. Normalization was achieved through logarithmic transformation and auto-scaling to unit variance. PCA and k-means clustering were used for unsupervised visualization of data structure and similarity among samples, whereas PLS-DA was applied to identify discriminant variables between experimental categories. VIP scores derived from PLS-DA were used to rank the contribution of each metabolite or compound feature to the observed clustering patterns. Hierarchical clustering and correlation heatmaps were generated based on Pearson correlation coefficients using Ward’s linkage method. Since the datasets were reconstructed from summarized values (means ± SEs), all statistical evaluations were strictly exploratory and descriptive; no hypothesis testing or *p*-value inference was performed.

### 4.6. Rationale and Reproducibility

This integrative analysis represents a data-mining approach applied exclusively to publicly available information derived from previously published work. The re-analysis aimed to provide a higher-level synthesis of metabolic and functional relationships without reprocessing raw instrumental data. All analytical steps are fully reproducible by any researcher with access to the published datasets, ensuring transparency, independence from the original experimental work, and methodological robustness.

## 5. Conclusions

This integrative cross-study metabolomic analysis revealed consistent associations between the chemical composition of food-derived extracts and their biological effects in a *Caenorhabditis elegans* model of Alzheimer’s disease. The combined use of multivariate statistics, functional bioassays, and chemical profiling enabled the identification of specific metabolite classes—such as polyphenols, flavonoids, and iridoids—linked to improved oxidative stress resistance, reduced β-amyloid-induced paralysis, and enhanced tau-associated motility. Notably, the clustering patterns and correlation networks derived from the data suggest that distinct extracts exhibit complementary strengths in chemical composition and neuroprotective action. While further validation is needed, these observations provide a foundation for proposing rational combinations of food matrices to maximize health-promoting potential through targeted biomedical strategies. Overall, this work highlights the value of integrating omics and bioactivity data to inform the design of next-generation nutritional interventions aimed at mitigating neurodegeneration and promoting healthy aging.

## Figures and Tables

**Table 1:** List of the origins of the materials employed in this study.

Food-Matrix Origin	Type	Reference
*Olea europaea*	Extract rich in oleuropein from leaves	[18]
*Olea europaea*	Olive-derived extract rich in hydroxytyrosol	[19]
*Olea europaea*	Olive leaf extracts with different contents of polyphenols	[20]
*Allium sativum*	Extract obtained from the white garlic variety BARI Roshun-1	[21]
*Brassica oleracea* L. var. *Italica* L.	A by-product extract from broccoli	[22]
*Fragaria × ananassa* cv. *Romina*	Methanolic extract from strawberry	[23]
*Tulbaghia violacea*	An extract synthesized from the petals of the edible flower	[25]
*Leptospermum scoparium*	Manuka honey	[27]
*Persea americana* Mill	Avocado honey	[28]

## Data Availability

The datasets analyzed in this study were extracted from the previously published articles cited in Table 1 [18,19,20,21,22,23,25,27,28]. The harmonized matrix and MetaboAnalyst workflow generated for this integrative re-analysis are available from the corresponding author upon reasonable request.

## Abbreviations

The following abbreviations are used in this manuscript:

ABTS	2,2′-azinobis(3-ethylenbenzothiazoline-6-sulfonic acid)
AChE IC_50_	Acetylcholinesterase inhibitory activity
AD	Alzheimer’s disease
COX	Cyclooxygenase
DCFDA	Dichlorodihydrofluorescein diacetate
DPPH	1,1-diphenyl-2-picrylhydrazyl
FRAP	Ferric reducing antioxidant power
GSH	Reduced glutathione
HCA	Hierarchical clustering analysis
HT	Hydroxytyrosol
H30	Hour 30
H32	Hour 32
OLE	Oleuropein
PC	Principal component
PCA	Principal Component Analysis
PLS-DA	Partial Least Squares Discriminant Analysis
p-Tau	Phosphorylated tau protein
ROS	Reactive oxygen species
VIP	Variable Importance in Projection
